# Association Between County-Level Natality and Income in the US, 2000-2020

**DOI:** 10.1001/jamapediatrics.2022.4814

**Published:** 2022-12-12

**Authors:** Nick Turner, Kendra Robbins

**Affiliations:** 1 Federal Reserve Board of Governors, Washington, DC; 2 Yale University, New Haven, Connecticut

## Abstract

**Importance:**

It is generally accepted that birth rates are negatively associated with income. However, less is known about the nature and evolution of natality differences between high- and low-income counties and how these differences are associated with the recent decline in the birth rate overall in the US.

**Objective:**

To quantify the association between county-level income and natality between 2000 and 2020 and explore how natality inequality is associated with recent nationwide natality declines.

**Design, Setting, and Participants:**

This cross-sectional study included all births to US women aged 15 to 44 years among county-year–level observations with at least 100 women of the same age.

**Exposures:**

County-level median household income rank.

**Main Outcomes and Measures:**

Association between natality and income and between natality inequality and national natality trends. Income ventiles were used to rank counties from 1 to 20, with each ventile including 5% of the female population aged 15 to 44 years. A counterfactual simulation was used to estimate natality rates while holding natality inequality constant at its year 2000 value.

**Results:**

A total of 86 679 356 births were aggregated to 65 554 county-year–level observations from 2000 to 2020. The analysis yielded 2 main results. First was the changing nature of inequality. Estimates of the natality income gradient, which reflect the association between county-level natality and county-level income, changed from −0.061 (95% CI, −0.200 to 0.078) in 2000 to −0.572 (95% CI, −0.678 to −0.466) in 2020, reflecting an increase in natality inequality. Intuitively, a negative gradient reflects that natality is lower in higher-income counties compared with lower-income counties. Second, the counterfactual simulation showed that there would have been an additional 3.5 million births (an increase of 4.1%) over the remainder of the study period had natality inequality remained constant at its year 2000 value. While this counterfactual is based on strong assumptions that are unlikely to hold in practice, it suggests that natality may have been higher during this period absent the rise in natality inequality.

**Conclusions and Relevance:**

The findings of this cross-sectional study suggest that natality inequality has increased in recent years and is likely associated with nationwide natality declines. The causes of this inequality remain to be explored in future work.

## Key Points

**Question:**

How does natality differ between high- and low-income counties in the US, and how are these differences associated with the nationwide natality decline in recent years?

**Findings:**

This cross-sectional study of 86 679 356 births between 2000 and 2020 presents 2 main findings. First, natality inequality increased from the mid-2000s through 2020, and second, counterfactual simulation suggested that this increased natality inequality is associated with national natality declines in recent years.

**Meaning:**

These findings suggest that natality inequality increased between the mid-2000s and 2020; this inequality may be associated with the overall US natality declines in recent years.

## Teaser

This cross-sectional study uses national agency data to evaluate the association between natality and income and explore how natality inequality is associated with declines in US birth rates.

## Introduction

In 2020, the birth rate in the US fell for the sixth consecutive year, and women had the fewest number of births since 1979.^1^ Although the COVID-19 pandemic likely affected the birth rate in 2020,^3^ the general reasons for the extended decline are not well understood.^4^ Against this backdrop, researchers have documented rising income inequality in the US.^5^ Although it is generally accepted that birth rates are negatively associated with income,^7^ less is known about the nature and evolution of natality differences between individuals with high and low incomes and how these differences are associated with the decline in the birth rate overall.

In this study, we aimed to provide a new perspective on the association between income and natality in the US from 2000 to 2020 by first quantifying natality inequality in each year and then highlighting the association between increasing natality inequality and the national natality decline in recent years through the use of a counterfactual simulation.

## Methods

This cross-sectional study used county-level natality and county-level income ranking in the national income distribution to quantify natality differences between lower- and higher-income counties. To estimate this association, we defined natality as the number of births to women aged 15 to 44 years per 1000 women of the same ages using data from the Centers for Disease Control and Prevention aggregated to mothers’ county of residence. For each year, we used US Census Bureau data to assign counties to income ventiles (20 groups of counties with equal female populations aged 15-44 years), ranked 1 to 20 by median household income. To quantify each county’s income rank in the national distribution, we used median household income, which is more likely to reflect household resources than individual-based income measures^9^ and has been shown to be associated with physical and mental health.^10^ Information on data sources is available in the eAppendix in the Supplement.

The sample included 86 679 356 births to women aged 15 to 44 years from 2000 to 2020 aggregated to 65 554 county-year–level observations that have at least 100 women. Sample means of natality and income for each income ventile in select years are shown in eTable 1 in the Supplement. This study analyzes publicly available information and therefore does not require ethical review under the Common Rule. This study followed the Strengthening the Reporting of Observational Studies in Epidemiology (STROBE) reporting guideline.

### Statistical Analysis

To quantify the association between natality and income rank, we estimated the following Equation, using ordinary least squares weighted by the female population aged 15 to 44 years:

*Natality_ct_* = β*_t_* × *IncomeRank_ct_ +* α*_t_ +* ε*_ct_*,

where *c* denotes county and *t* indexes year. This specification is based on earlier work examining mortality inequality.^13^ The parameter β represents the association between natality and income rank (1-20), which we refer to as the natality-income gradient. This gradient measures the change in natality per 1-unit increase in the income ventile distribution (increasing 5 percentile points in the national income distribution ranking). A gradient equal to 0 would reflect perfect equality, characterized by no change in natality across the income distribution. Nonzero values reflect deviations from equality. For example, a negative value reflects fewer births in higher-income counties. The parameter α represents the intercept term, and ε is the error term. We used Stata/MP, version 16.1 software (StataCorp LLC) to calculate estimates with robust SEs.^17^ We interpreted estimation results as statistically significant based on 2-tailed tests at the 5% level.

To illustrate the association between changing natality inequality and national natality trends, we constructed a counterfactual scenario that holds inequality fixed over time, an approach that follows related work on infant mortality inequality in the US.^15^ We defined counterfactual natality as the nationwide natality that would have occurred if the natality-income gradient did not change over the sample period but instead remained at its value from the year 2000 over the next 20 years. This counterfactual scenario is based on 2 strong assumptions: (1) that natality inequality remains constant over time and (2) that constant inequality does not affect the evolution of other factors associated with natality. In other words, only the evolution of natality inequality changes in the counterfactual scenario, and all other factors follow their actual patterns over time.

We did not interpret the counterfactual scenario as a probable outcome. Instead, it serves as a helpful benchmark to highlight how natality inequality compares with overall national trends. Intuitively, the counterfactual is based on the following thought experiment: How would national natality have evolved if natality inequality remained fixed at the 2000 level throughout the remainder of the sample period (and all other factors are unchanged)? Differences between counterfactual and actual natality result only from differences in the evolution of the gradient over time, helping to illustrate how changes in inequality are associated with overall natality trends.

We impute counterfactual natality using parameter estimates from the Equation by combining year-specific intercept terms with the estimate of the natality-income gradient (our proxy for inequality) from 2000. Specifically, counterfactual natality for all years *t*≥2000 is equal to the year *t* intercept (α*_t_*) plus the estimated 2000 gradient multiplied by the year *t* household income ventile (β*_2000_* × *IncomeRank_ct_*). Using the estimated gradient from the year 2000, the counterfactual holds fixed the level of natality inequality over time. The use of the year-specific intercept reflects the assumption that the evolution of other factors remains the same between actual and counterfactual natality.

## Results

**Figure 1.**
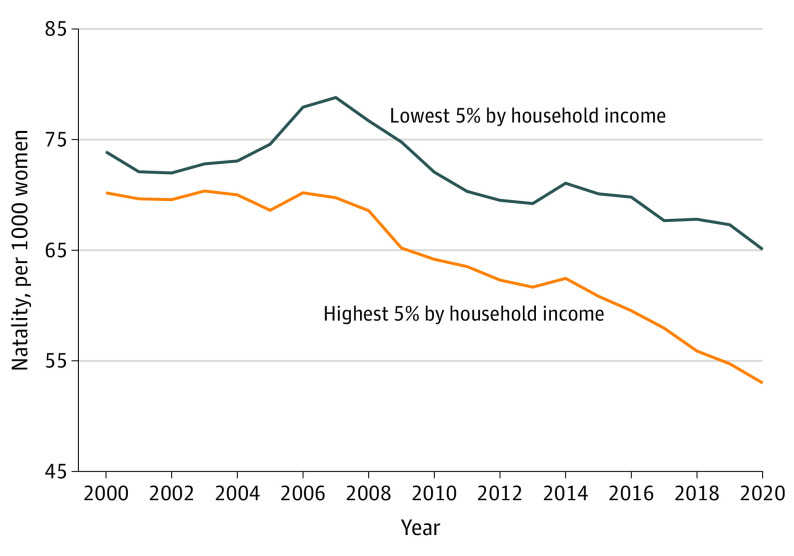
Natality for the Highest- and Lowest-Income 5% of US Counties, 2000-2020 Income groups are defined based on aggregations of counties (weighted by female population aged 15-44 years), and natality is defined per 1000 women aged 15-44 years. Data on natality are from the Centers for Disease Control and Prevention, and population and income data are from the US Census Bureau.

As shown in Figure 1, the difference in natality between the highest income ventile and the lowest income ventile was relatively constant between 2000 and 2004. In contrast, between 2005 and 2020, this difference generally widened each year, with the largest difference in 2020. Natality decreased during this period for both the highest- and lowest-income counties, but the decline was relatively larger in the highest-income counties. Overall, between 2000 and 2020, the percent decrease in natality was approximately twice as large in the highest income ventile compared with the lowest income ventile.

**Figure 2.**
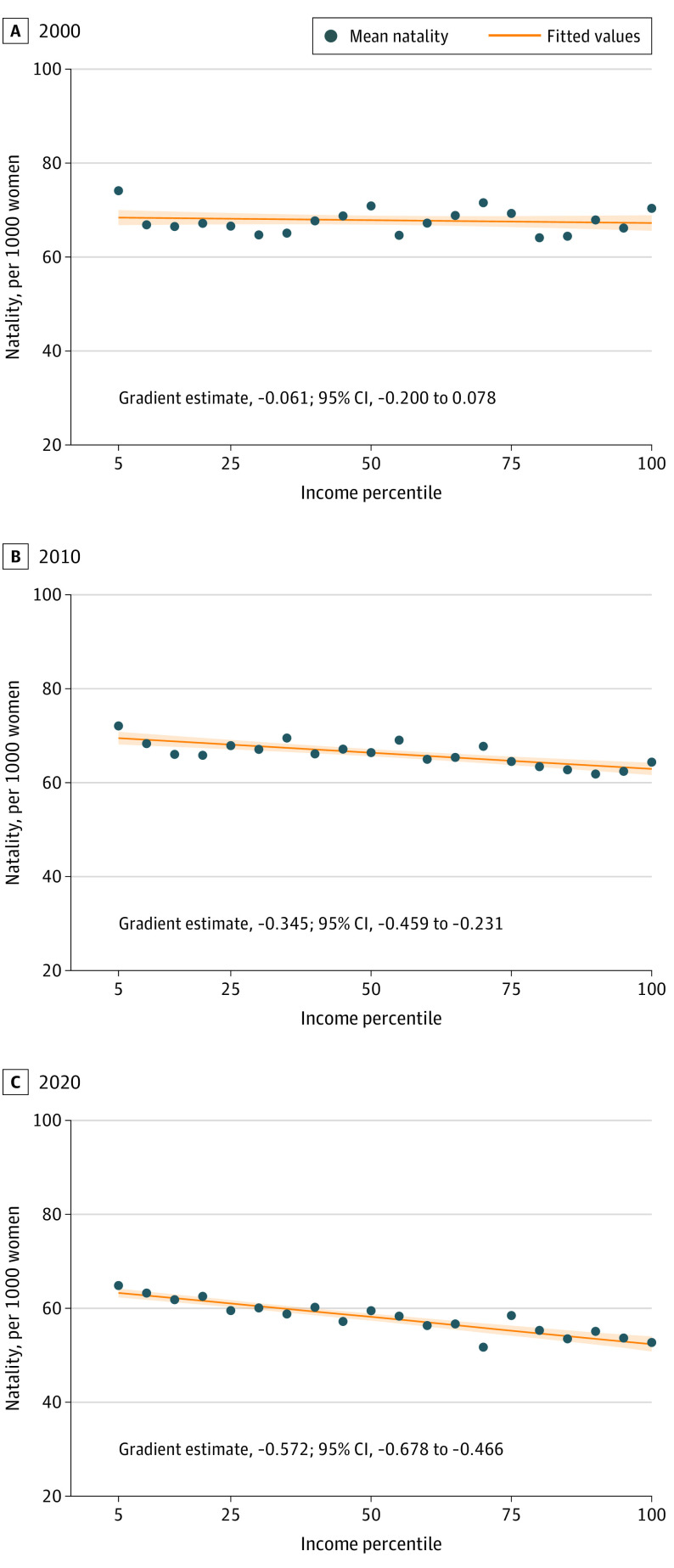
Natality and Fitted Values, 2000, 2010, and 2020 Shown are the mean natality by income percentile for each income ventile, fitted values estimated from the Equation, and 95% CI of the fitted value. Data on natality are from the Centers for Disease Control and Prevention, and income data are from the US Census Bureau.

**Figure 3.**
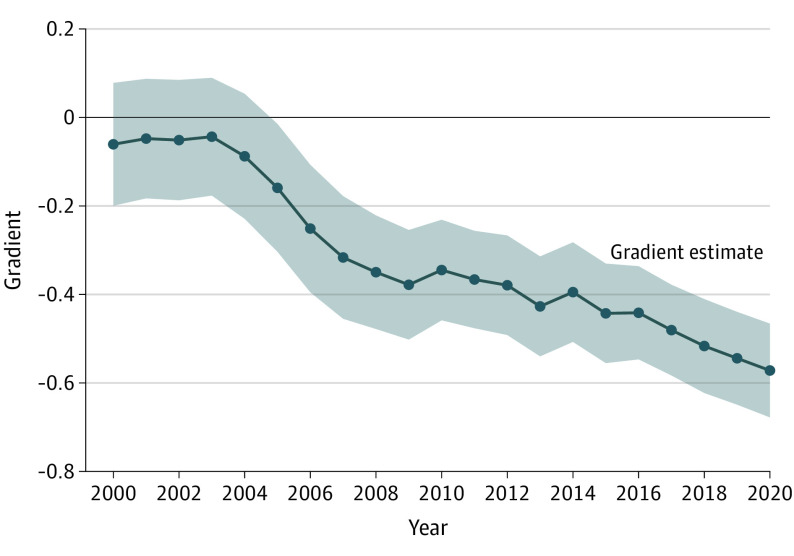
Natality-Income Gradient, 2000-2020 Shown are point estimates of the natality-income gradient estimated using the Equation. The 95% CIs (shaded area) are based on 2-tailed tests using robust SEs. Data on natality are from the Centers for Disease Control and Prevention, and income data are from the US Census Bureau.

Figure 2 and Figure 3 characterize the association between natality and income rank over the entire income distribution. Figure 2 shows the natality-income gradients for 2000, 2010, and 2020, including the mean natality by income ventile, fitted values from the Equation, and the 95% CI of the fitted values. The natality-income gradient is the slope of the solid orange line; gradient estimates for all years are shown in eTable 2 in the Supplement. The gradient estimates shown in Figure 2 suggest that natality is well approximated using a linear function of income rank. Figure 2 also implies that the association between natality and income changes over time during the sample period, moving from a relatively flat gradient in 2000 (gradient estimate, −0.061; 95% CI, −0.200 to 0.078) (Figure 2A) to a more negative association in later years (gradient estimate, −0.345 [95% CI, −0.459 to −0.231] for 2010 and −0.572 [95% CI, −0.678 to −0.466] for 2020) (Figure 2B and C).

To explore how the gradient changed across all years in the sample period, Figure 3 shows estimates of the natality-income gradient and 95% CIs for every year, highlighting that natality transitions from equality in 2000 to 2004 (gradient not statistically different from 0) to increasing inequality (gradient is negative and statistically different from 0) during later years in the sample period. In 2005, the gradient is statistically different from 0 and negative, reflecting relatively lower birth rates in high-income counties compared with low-income counties. Thereafter, the gradient becomes more negative in later years, reflecting widening natality inequality, reaching the most unequal gradient in the sample period in 2020. The gradient estimate in 2020 suggests that natality decreased 0.572 (estimate, −0.572; 95% CI, −0.678 to −0.466; *P* < .001) per 1-ventile increase in the income distribution (increasing 5 percentile points in the national income distribution scale).

**Figure 4.**
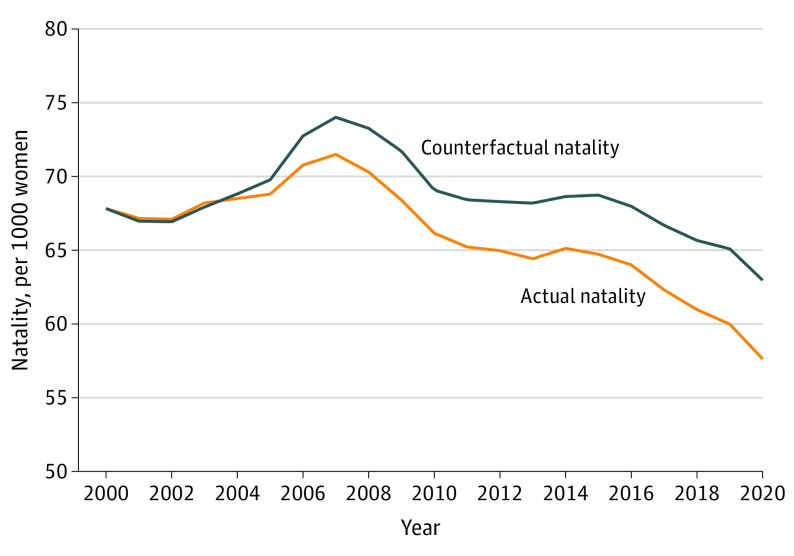
Actual and Counterfactual Natality, 2000-2020 The counterfactual natality scenario holds fixed the natality-income gradient from the year 2000 value in all future years. Data on natality are from the Centers for Disease Control and Prevention, and income data are from the US Census Bureau.

To answer the question of how natality would have evolved nationally during the sample period if natality inequality had not increased (and all other factors were unchanged), we compared actual natality and counterfactual natality (Figure 4). The counterfactual scenario holds fixed the natality-income gradient from the year 2000 value over the following 20 years. In 2020, actual natality was lower than the counterfactual scenario, suggesting that if natality inequality had not increased after 2000, natality for the nation would likely have been higher throughout the sample period. In the counterfactual scenario, the decline in national natality is 4.6 births per 1000 women between 2000 and 2020, less than half as large as the actual decline of 10.2 births per 1000 women (eTable 3 in the Supplement shows actual and counterfactual natality used to calculate the declines). Translating the natality differences into births across the entire 20-year period from 2001 to 2020, counterfactual natality implies an additional 3.5 million births (an increase of 4.1%). However, the counterfactual simulation is based on strong assumptions that are unlikely to hold in practice, so the exact numerical results should be interpreted with caution. Despite this limitation, the patterns shown in Figure 4 suggest that overall national declines in natality would likely have been smaller absent the rise in natality inequality in recent years (assuming that other factors associated with natality inequality were also unchanged).

We confirmed that the main results are not sensitive to alternate assumptions in several ways. First, as shown in eFigure 1 in the Supplement, the main implications from Figure 2 still hold when using the level of median household income instead of the income rank (the gradient in eFigure 1 in the Supplement represents the association between natality and an additional $1000 of median income). In our baseline specification, we used income rank for several reasons, including comparability with related literature,^13^ and to focus on relative income differences. Second, the results were not sensitive to alternate sample selections based on the population of women in a county (the baseline results include county-year–level observations with at least 100 women to mitigate the effect of outlying observations resulting from small populations). As shown in eFigure 2 in the Supplement, the results are virtually identical when including only counties that meet the population threshold in all years or when not imposing a population threshold. Third, as shown in eTable 3 in the Supplement, the implications from the counterfactual scenario also hold when using 2010 as a base year to define the counterfactual level of inequality. Of note, these implications continue to hold when using the 2010 level of inequality that is higher than the 2000 level used in the baseline analysis and when the counterfactual scenario is projected over a shorter period.

## Discussion

In this cross-sectional study, our findings suggest that natality inequality began to increase steadily in 2005, reaching the most unequal level at the end of the sample period in 2020. This widening natality gap between high- and low-income counties may have a meaningful association with overall national natality trends. Results from our counterfactual simulation suggest that natality could have been more than 4% higher overall between 2001 and 2020 if natality inequality had not increased.

### Limitations

This study has several limitations. First, our analyses of county-level natality-income gradients do not represent comparisons of high-income vs low-income individuals but rather the association between natality and residing in a higher-income county compared with a lower-income county. Second, our estimates cannot be interpreted as causal effects, as median county income is likely associated with other factors that affect natality. Third, our measure uses variation across counties but excludes variation within counties. Fourth, our data collection period ended in 2020, a year in which the COVID-19 pandemic may have contributed to fertility decisions. Preliminary aggregate data showed a notable rise in natality in 2021.^18^ Evaluating the underlying reasons for this increase and the extent to which there was a shift in the natality-income gradient are outside the scope of this article but remain open questions for future work when disaggregated data become available. Fifth, although our counterfactual simulation helps to illustrate the association between inequality changes and national natality trends, it is based on strong assumptions that are unlikely to hold in practice, and exact numerical results should be interpreted with caution in this context.

### Conclusion

 The findings of this cross-sectional study suggest that natality inequality increased in recent years and is likely associated with nationwide natality declines. Understanding the forces driving natality inequality, characterizing the relationship between natality inequality and shifts in the maternal age distribution,^19^ and how the COVID-19 pandemic affected this relationship remain open questions for future work.

## Notes

Supplement. **eAppendix.** Data Sources **eTable 1.** Natality and Income by Ventile, Select Years **eTable 2.** Natality-Income Gradient Estimates, 2000-2020 **eTable 3.** Actual and Counterfactual National Natality, 2000-2020 **eFigure 1.** Alternate Natality-Income Gradient, 2000-2020 **eFigure 2.** Natality-Income Gradient, Alternate Sample Restrictions, 2000-2020
