# Supplementary material for: Association Between County-Level Natality and Income in the US, 2000-2020
Source: JAMA Pediatr. 2022 Dec 12:e224814. Online ahead of print. doi: 10.1001/jamapediatrics.2022.4814 (PMC9857058; doi:10.1001/jamapediatrics.2022.4814)
Supplement: Supplement. — eAppendix. Data Sources eTable 1. Natality and Income by Ventile, Select Years eTable 2. Natality-Income Gradient Estimates, 2000-2020 eTable 3. Actual and Counterfactual National Natality, 2000-2020 eFigure 1. Alternate Natality-Income Gradient, 2000-2020 eFigure 2. Natality-Income Gradient, Alternate Sample Restrictions, 2000-2020 [file jamapediatr-e224814-s001.pdf]

Supplemental Online Content

Turner N, Robbins K. Association between county-level natality and income in the US, 2000-2020. *JAMA Pediatr*. Published online December 12, 2022. doi:10.1001/jamapediatrics.2022.4814

eAppendix. Data Sources

eTable 1. Natality and Income by Ventile, Select Years

eTable 2. Natality-Income Gradient Estimates, 2000-2020

eTable 3. Actual and Counterfactual National Natality, 2000-2020

eFigure 1. Alternate Natality-Income Gradient, 2000-2020

eFigure 2. Natality-Income Gradient, Alternate Sample Restrictions, 2000-2020

This supplemental material has been provided by the authors to give readers additional information about their work.

© 2022 Turner N et al. *JAMA Pediatrics*.

eAppendix. Data Sources

Researchers can access data using the sources described below. Restricted-use natality data requires approval by the Centers for Disease Control and Prevention (CDC). Population and poverty data from the U.S. Census Bureau are publicly available.

Microdata containing all U.S. births from 2000 to 2020 were obtained from the CDC National Vital Statistics System and were used to calculate county-level natality rates. Information about the data application process is available at https://www.cdc.gov/nchs/nvss/nvss-restricted-data.htm.

County-level median household income data were obtained from the Census Bureau Small Area Income and Poverty Estimates Program (SAIPE) for years 2000-2020. These data are publicly available at https://www.census.gov/programs-surveys/saipe/data/datasets.html.

County-level population data by age and gender were obtained from the U.S. Census Bureau. These files are available here: https://seer.cancer.gov/popdata/download.html

© 2022 Turner N et al. *JAMA Pediatrics*.

eTable 1. Natality and Income by Ventile, Select Years

(1)	(2)	(3)	(4)	(5)	(6)	(7)
Income Ventile	Natality (per 1,000)			Median Income (thousands, 2020 dollars)		
	2000	2010	2020	2000	2010	2020
1	74.1	72.1	64.8	40.9	38.6	42.6
2	66.9	68.3	63.2	46.4	43.5	48.2
3	66.5	66.0	61.8	49.6	46.2	51.5
4	67.2	65.8	62.5	52.2	47.9	53.8
5	66.6	67.9	59.5	54.4	49.5	55.7
6	64.7	67.1	60.1	56.4	50.7	57.7
7	65.1	69.5	59.0	58.4	52.5	59.8
8	67.7	66.1	60.0	60.0	54.1	61.8
9	68.8	67.1	57.2	61.5	55.7	63.5
10	70.9	66.4	59.4	62.8	57.3	65.4
11	64.6	69.1	58.3	64.1	59.6	67.2
12	67.2	65.0	56.3	65.8	60.9	70.5
13	68.9	65.4	56.6	67.9	62.2	72.0
14	71.6	67.7	51.6	70.6	63.8	74.8
15	69.3	64.5	58.4	72.7	67.3	77.8
16	64.1	63.4	55.2	76.0	71.0	81.4
17	64.4	62.7	53.5	81.8	75.5	86.2
18	67.9	61.8	54.9	86.8	81.8	91.3
19	66.2	62.4	53.7	95.2	88.9	100.9
20	70.4	64.4	52.8	108.9	103.9	120.5

Notes: Income ventiles classify counties into 20 equally sized groups (based on female population ages 15-144), ranked by income. Natality is defined as births to women ages 15-44 per 1,000 women ages 15-44. Income is adjusted to 2020 dollars using the Consumer Price Index (CPI). Natality data are from the CDC, income and population data are from the U.S. Census Bureau, and CPI data are from the Bureau of Labor Statistics.

© 2022 Turner N et al. *JAMA Pediatrics*.

eTable 2. Natality-Income Gradient Estimates, 2000-2020

(1)	(2)	(3)	(4)	(5)	(6)
**Year**	**Natality-Income Gradient**	**Standard error**	**Constant**	**Standard error**	**Observations**
2000	-0.061	[0.071]	68.459***	[0.878]	3,125
2001	-0.048	[0.069]	67.665***	[0.844]	3,124
2002	-0.051	[0.069]	67.632***	[0.865]	3,125
2003	-0.044	[0.068]	68.649***	[0.852]	3,128
2004	-0.088	[0.072]	69.410***	[0.904]	3,127
2005	-0.159**	[0.074]	70.483***	[0.942]	3,128
2006	-0.251***	[0.073]	73.438***	[0.861]	3,124
2007	-0.316***	[0.071]	74.870***	[0.846]	3,121
2008	-0.350***	[0.065]	74.041***	[0.797]	3,118
2009	-0.378***	[0.063]	72.445***	[0.804]	3,117
2010	-0.345***	[0.058]	69.827***	[0.732]	3,117
2011	-0.366***	[0.056]	69.100***	[0.683]	3,117
2012	-0.379***	[0.057]	68.978***	[0.714]	3,118
2013	-0.427***	[0.058]	68.942***	[0.686]	3,120
2014	-0.395***	[0.057]	69.304***	[0.685]	3,122
2015	-0.443***	[0.057]	69.436***	[0.665]	3,120
2016	-0.441***	[0.054]	68.712***	[0.628]	3,120
2017	-0.481***	[0.052]	67.444***	[0.585]	3,122
2018	-0.517***	[0.054]	66.528***	[0.571]	3,120
2019	-0.544***	[0.053]	65.859***	[0.564]	3,122
2020	-0.572***	[0.054]	63.821***	[0.534]	3,119

Notes: Column (2) shows estimates of the natality-income gradient with statistical significance using two-tailed tests noted as: * <0.1, **<0.05, *** <0.01. Column (3) reports robust standard errors of the gradient estimates. Column (4) shows estimates of the intercept term, and Column (5) reports robust standard errors of the intercept. Column (6) shows the number of observations used in each estimation of Equation (1). Natality data are from the CDC, population and income data are from the U.S. Census Bureau.

© 2022 Turner N et al. *JAMA Pediatrics*.

eTable 3. Actual and Counterfactual National Natality, 2000-2020

(1)<br>Year	(2)<br>Actual natality	(3)<br>2000 Counterfactual natality	(4)<br>2010 Counterfactual natality
2000	67.8	67.8	--
2001	67.2	67.0	--
2002	67.1	67.0	--
2003	68.2	68.0	--
2004	68.5	68.8	--
2005	68.8	69.8	--
2006	70.8	72.8	--
2007	71.5	74.2	--
2008	70.3	73.4	--
2009	68.4	71.8	--
2010	66.2	69.2	66.2
2011	65.2	68.5	65.4
2012	65.0	68.3	65.3
2013	64.4	68.3	65.3
2014	65.1	68.7	65.7
2015	64.7	68.8	65.8
2016	64.0	68.1	65.0
2017	62.3	66.8	63.8
2018	61.0	65.9	62.8
2019	60.0	65.2	62.1
2020	57.6	63.2	60.1

Notes: In all columns, natality is defined as births to women ages 15-44 per 1,000 women ages 15-44. Counterfactual fertility rates (columns (2)-(5)) are calculated using parameters from Equation (1). Each counterfactual fertility rate is calculated using time-varying intercept terms for the year given by column (1) combined with the time-invariant gradient estimate noted in the column heading. For example, the 2000 counterfactual for years $t \geq 2000$ is calculated as the year t intercept term plus the product of the year t income rank and the year 2000 estimated gradient. eTable 2 shows the gradient and intercept estimates.

© 2022 Turner N et al. *JAMA Pediatrics*.

eFigure 1. Alternate Natality-Income Gradient, 2000-2020

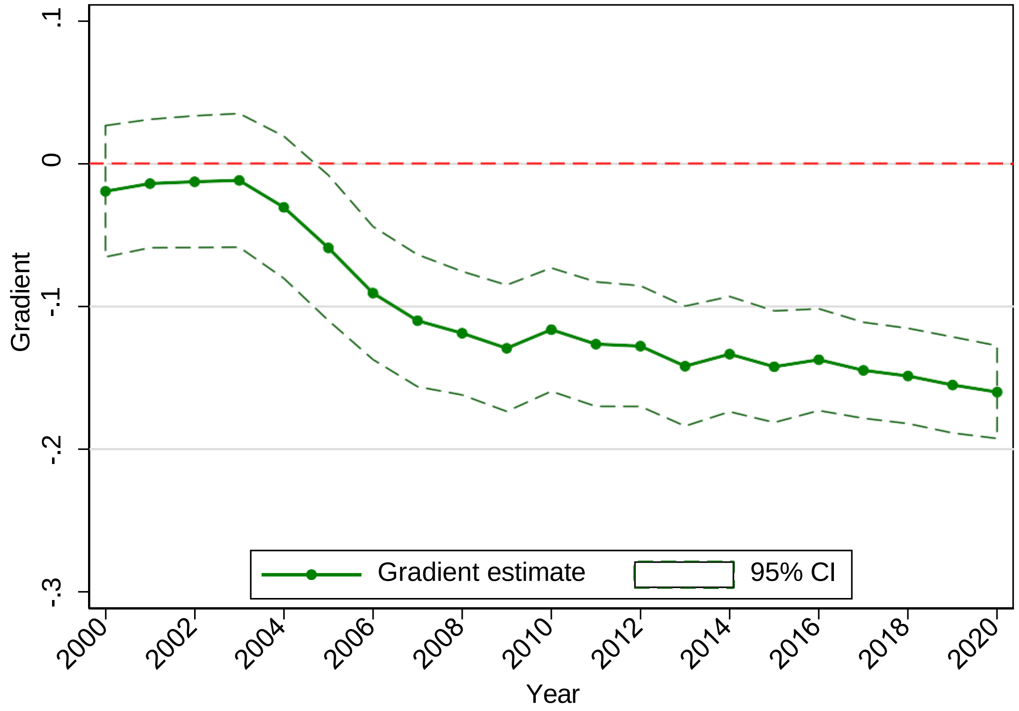

Notes: This figure plots the gradient and standard errors of the natality-median income level gradient, where median income is measured in thousands of constant dollars (2020). The green circles denote point estimates of the natality-income gradient. The dashed green lines show the 95% confidence intervals based on two tailed tests using robust standard errors. Data on natality are from the CDC, and income data are from the U.S. Census Bureau.

© 2022 Turner N et al. *JAMA Pediatrics*.

eFigure 2. Natality-Income Gradient, Alternate Sample Restrictions, 2000-2020

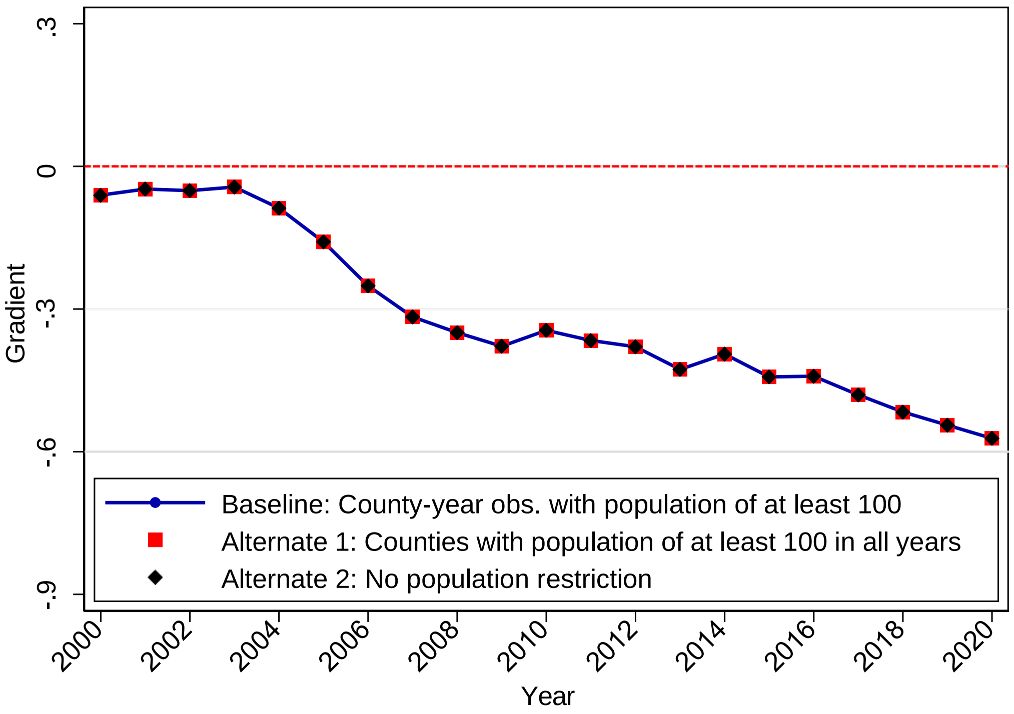

Notes: This figure plots the natality-income gradient for three different samples: (1) the baseline sample that includes only county-year level observations with at least 100 women ages 15-44 shown in blue line, (2) the set of counties that have at least 100 women ages 15-44 in each year from 2000 to 2020 shown in red squares, (3) the sample with no population restrictions shown in black diamonds. Data on natality are from the CDC, and income data are from the U.S. Census Bureau.

© 2022 Turner N et al. *JAMA Pediatrics*.
